# Protein localization and potential function of lipocalin in *Reticulitermes speratus* queens

**DOI:** 10.1371/journal.pone.0311836

**Published:** 2024-10-07

**Authors:** Takumi Hanada, Anji Kobayash, Hajime Yaguchi, Kiyoto Maekawa

**Affiliations:** 1 Graduate School of Science and Engineering, University of Toyama, Gofuku, Toyama, Japan; 2 Department of Forest Entomology, Forestry and Forest Products Research Institute, Tsukuba, Ibaraki, Japan; 3 Academic Assembly, University of Toyama, Gofuku, Toyama, Japan; Laboratoire de Biologie du Développement de Villefranche-sur-Mer, FRANCE

## Abstract

To understand the mechanisms underlying social evolution and caste development in social insects, caste-specific organs and genes should be investigated. In the rhinotermitid termite, *Reticulitermes speratus*, the lipocalin gene *RS008881*, which encodes a protein transporter, is expressed in the ovarian accessory glands of primary queens. To obtain additional data on its expression and product localization, we conducted real-time quantitative polymerase chain reaction and protein assays using a peptide antibody. Gene expression analysis of the castes revealed that *RS008881* was highly expressed in female primary and secondary reproductives. Further analysis of its expression during reproductive caste differentiation showed that its expression levels increased prior to molting into reproductive individuals, even during the winged imago (alates) stage. Western blotting and fluorescent immunohistochemical staining revealed that the RS008881 product was localized in the ovary as well as the eggshells produced by female reproductives. RS008881 may play a significant role in the reproductive biology of *R*. *speratus*; protein localization in both the ovary and eggshell suggests multiple functions related to embryo protection and potential pheromone interactions.

## Introduction

The division of labor among individuals for reproduction is the most important characteristic of eusociality. Recent advances in sociogenomics have enables the identification of several caste-specific genes in termites [1]. Among these differentially expressed genes (DEGs), those highly expressed in the reproductive caste are of particular interest because some of them may be related to the reproductive division of labor. Several genes highly expressed in the queen ovarian accessory glands of the rhinotermitid subterranean termite, *Reticulitermes speratus*, have been identified [2]. One of these proteins is lipocalin, which may play a crucial role in the reproductive functions of queens.

In termites, the ovarian accessory glands are usually located under the posterior sternites of female reproductives [3]. In the rhinotermitid species *Pseudacanthotermes spiniger*, the ovarian accessory glands are more complex and composed of multiple cells [4], differing from those found in cockroaches, which have single-cell glands. However, the function of the ovarian accessory glands remains unclear [5].

Genes belonging to the lipocalin family encode protein transporters that bind to small hydrophobic molecules such as pheromones [6]. The lipocalin family is known to have three conserved motifs (structurally conserved regions). However, the rapid evolutionary rates of these genes have resulted in significant amino acid sequence diversity [7]. Although specific ligands have not been identified for lipocalins in termites, some evidence suggests that lipocalins play essential roles in social maintenance. For example, in *Zootermopsis nevadensis*, the lipocalin homolog (*Neural Lazarillo*; *ZnNLaz1*) is highly expressed in soldier-destined individuals in the incipient colony, and it may regulate soldier differentiation via interaction with the queen [8]. In *Hodotermopsis japonica* (= *sjostedti*), a lipocalin gene (*SOL1*) is specifically expressed in the soldier mandibular glands and is probably involved in transmission of chemicals that regulate caste differentiation and/or defensive behavior [9, 10]. Moreover, in *R*. *speratus*, several pheromones derived from a queen have been identified [11, 12]. Consequently, lipocalin is highly expressed in the ovarian accessory glands and may interact with these pheromones as a protein transporter [2]. To determine the function of lipocalin, examining changes in gene expression during reproductive caste differentiation and protein localization in reproductive organs and eggs is crucial.

This study focused on the lipocalin gene *RS008881*, which has been confirmed to be expressed in the queen accessory glands in *R*. *speratus* [2]. First, we analyzed the expression levels of *RS008881* among the castes and at different developmental stages using real-time quantitative polymerase chain reaction (RT-qPCR). Next, using a peptide antibody against RS008881, we performed western blotting and fluorescence immunohistochemical staining to determine the localization of its product in the bodies of reproductives and eggs. Based on the results obtained, we discussed the potential role of RS008881 in *R*. *speratus*.

## Materials and methods

### Termites

Mature colonies of *R*. *speratus* were collected from decaying wood in Toyama Prefecture, Japan, between 2020 and 2022. The colonies were maintained in plastic containers in a moist environment under constant darkness at room temperature. Primary reproductives (kings and queens) were obtained from previously established incipient colonies as per the method described by [13]. The following castes and stages were examined: primary and secondary female reproductives (queens), female alates (winged imagoes), female nymphs, workers, soldiers, and eggs. To obtain the secondary queens (just after molting), individuals induced from the sixth-instar nymphs as per the method described in previous studies [14, 15] were used.

### RNA extraction and cDNA synthesis

To compare the expression levels of *RS008881* among castes, three individuals of primary reproductives (kings and queens, five months after colony foundation), workers, and soldiers of both sexes (n = 3) were collected, of which the head and thorax/abdomen were dissected. Three different biological replications were prepared from three different colonies. Samples were then immediately frozen in liquid nitrogen and stored at -80°C until RNA extraction. Total RNA extraction and genomic DNA removal were performed as per manufacturer’s instructions of the ReliaPrep RNA Miniprep System (Promega, WI, USA). The quantity and quality of the extracted total RNA were determined using a NanoVue spectrophotometer (GE Healthcare Life Sciences, Buckinghamshire, UK) and Qubit 2 fluorometer (Thermo Fisher Scientific, MA, USA). After pooling an equal amount of RNA from the head and thorax/abdomen (0.154 μg each), cDNA synthesis was performed using a High-Capacity cDNA Reverse Transcription Kit (Thermo Fisher Scientific).

To compare the expression levels of *RS008881* among individuals from different developmental stages of females, 6–12 female individuals each of the following stages were collected: [primary queen formation] last-instar nymphs (with non-swollen or fully developed wing buds), alates (just after the molt), and primary queens (at 6.5 months after colony foundation); [secondary queen formation] sixth-instar nymphs, secondary queens (within 24 h following molting), and physogastric secondary queens (collected from a colony). Alates and nymphs were collected from one mature colony and secondary physogastric queens were collected from another mature colony. The abdomen of each individual was dissected. Two different abdomens were pooled as one sample and 3–6 biological replications were prepared for each developmental stage. RNA extraction and cDNA synthesis were performed as previously described.

### Real-time quantitative PCR

Based on the *RS008881* sequences [2], specific primers were designed for RT-qPCR using Primer 3 Plus [16] (**Table 1**). Using the THUNDERBIRD SYBR qPCR Mix (TOYOBO, Osaka, Japan) or PowerTrack SYBR Green Master Mix (Thermo Fisher Scientific) RT-qPCR analysis was performed using the Mini Option Real-time PCR system (Bio-Rad, CA, USA) and QuantStudio3 (Thermo Fisher Scientific). Amplification of single products was confirmed via analyzing the dissociation curves and amplified fragment lengths. As internal reference genes, the following six genes were used as per previous studies [17, 18]: *EF-1* (Accession No. AB602838), *β-actin* (No. AB520714), *NADH-dh* (no. AB602837), *GstD1* (gene ID: RS001168), *EIF-1* (RS005199), and *RPS18* (RS015150). The optimal internal reference gene was selected using GeNorm [19] and NormFinder [20]. Relative expression levels of *RS008881* were calculated by adopting the standard curve method using QuantStudio Design & Analysis Software v1.5 (Thermo Fisher Scientific). To compare expression levels among different castes, a two-way analysis of variance (ANOVA) was performed, followed by multiple comparisons using Tukey’s test. To compare expression levels among female individuals at different developmental stages, one-way ANOVA was performed followed by the Tukey-Kramer test. These analyses were performed using the Mac Statistical Analysis ver. 2.0 and 3.0 (Esumi, Tokyo, Japan).

**Table 1:** Primer sequences used in this study.

Gene symbol	Accession no/Gene ID	Forward sequence (5’ - 3’)	Reverse sequence (5’ - 3’)
*RS008881*	RS008881	CAAGCCGCTGTTTGTAGTCG	TTGGCCACGGAATACCAGTC
*EF-1*	AB602838	GGTGATGCGGCTATTGTTAACC	GTGGTGGGAATTCTGAGAAAGATT
*β-Actin*	AB520714	AGCGGGAAATCGTCCGTGAC	CAATGGTGATGACCTGCCCAT
*NADH-dh*	AB602837	GCTGGGGGGGTTATTCATTCCAT	GGCATACCACAAAGGGCAAAA
*GstD1*	RS001168	GCTGTTGGTGTGGATTTGAA	GTATGCTGCGGGTTCATCTT
*EIF-1*	RS005199	ATGGTAGGCTTGAAGCGATG	TTTGCATCCTGGTAGTCACG
*RPS18*	RS015150	ACTCTCAGCTCACATCCAGT	CCTCAGGCCCCAATAATGTC

### Antibody production

Based on the predicted RS008881 protein structure, hydrophilicity plot (Kyte-Doolittle), antigenic index (Jameson-Wolf), and surface probability plot (Emini), Hokudo (Sapporo, Japan) proposed three candidate antigen regions. By comparing the protein sequences of lipocalins identified in *R*. *speratus* [2], the region of RS008881 with the highest specificity (with at least two amino acid differences compared to other genes) was selected as the antigen site. The rabbit polyclonal antibody specific to RS008881 (synthetic peptide sequence: Cys-SFKFTKDDTWHNVI-OH) was produced by Hokudo. The antibody was evaluated using the enzyme-linked immunosorbent assay (ELISA) method described by Hokudo and subsequently subjected to Protein A purification. Affinity purification was performed using a HiTrapTM NHS-activated HP column (1 ml) (GE Healthcare Life Sciences). For the coupling reaction during column washing, a ligand solution containing a free peptide solution (4.93 mg/2 ml phosphate-buffered saline) was used. After the coupling reaction, blocking and equilibration were performed to create a ligand-immobilized column. The resulting RS008881 antibody (3.3 mg/ml) was added to the ligand-immobilized column (1 ml), after which elution and neutralization were performed to obtain the affinity-purified antibody (1.07 mg/ml). The prepared antibody was stored at -20°C until following experiments.

### Western blotting

Five physogastric secondary queens, workers, soldiers, and egg masses were collected. Each sample was immediately frozen in liquid nitrogen and stored at -80°C. The tissue was homogenized in tubes using a homogenizer pestle (AS ONE, Osaka, Japan) and protein extraction was performed using the EzRIPA Lysis kit (ATTO, Tokyo, Japan). Additionally, egg masses (approximately 25 mg) were collected from mature colonies and crushed using a homogenizer and pestle to separate eggshells from the liquid components containing embryos. Protein was extracted from each fraction as described previously.

Extracted proteins were processed using EzApply (ATTO) and subjected to sodium dodecyl sulfate (SDS)-polyacrylamide gel electrophoresis (PAGE). The protein molecular weight marker used was AE-1450 EzStandard PrestainBlue (ATTO). After electrophoresis, the gel was transferred onto a polyvinylidene fluoride (PVDF) membrane (Bio-Rad) using the semi-dry blotting technique with EzBlot (ATTO). Following transfer, the PVDF membrane was washed and blocked with a blocking solution (0.3% skim milk/tris-buffered saline with 0.1% Tween® 20; TBS-T). The primary rabbit polyclonal antibody explained above (after affinity purification, 1.07 mg/ml) was diluted 1/1000 in the blocking solution and the membrane was incubated with the primary antibody. After washing, the secondary antibody, goat anti-rabbit IgG (H + L) and Horseradish Peroxidase (HRP) conjugate (Proteintech, IL, USA), was diluted 1/5000 in the blocking solution and incubated with the membrane to detect the target protein using EzWestBlue or EzWestBlue W (ATTO). The images were captured by the Dolphin-doc (Kurabo, Osaka, Japan).

To analyze proteins extracted from the eggshell or liquid fractions, total protein was detected using EzStain Aqua MEM (ATTO) before the blocking step. Subsequently, western blotting was performed from the blocking step to detecting the target protein bands as per manufacturer’s protocol. The detected images were analyzed using ImageJ (National Institutes of Health, MD, USA) to calculate the intensity values of both the total and target proteins. Normalization was performed according to the protocol used to quantify signal strength.

### Fluorescent immunohistochemistry

Primary queens were collected from incipient colonies (approximately six months after colony foundation). Physogastric secondary queens, female last-instar nymphs, and egg masses were collected from mature colonies. Each sample was fixed in a 4% paraformaldehyde-phosphate buffer (Wako, Tokyo, Japan). Preserved samples were sequentially transferred to 10, 20, and 30% sucrose/phosphate-buffered saline solutions, embedded in Tissue-Tek O.C.T. compound (Sakura Finetek, Tokyo, Japan), and frozen in liquid nitrogen. Frozen sections were prepared using a CM1510S cryostat (Leica, Wetzlar, Germany) and mounted on crest-prevention-coated glass slides (Matsunami Glass, Osaka, Japan).

Prepared sections were washed with TBS-T and blocked at room temperature for 30 min with BBT solution (0.2% bovine serum albumin/TBS-T). Subsequently, they were again blocked for 30 min with 2% normal goat serum/BBT solution. The sections were then incubated with the primary antibody solution (after affinity purification, 1.07 mg/ml), which was prepared at a 1/500 dilution in 2% normal goat serum/BBT solution and incubated at 4°C overnight (approximately 12 h) for the primary antibody reaction. As a control, 2% normal goat serum/BBT solution (solvent for primary antibody solution) was used instead of the primary antibody solution. Following the reaction, sections were incubated with BBT and blocked at room temperature for 30 min with 2% normal goat serum/BBT solution. The sections were then incubated with the secondary antibody solution, which was prepared at a 1/200 dilution in 2% normal goat serum/BBT solution and incubated at room temperature in the dark for 2 h for the secondary antibody reaction. For the secondary antibody, the Goat anti-Rabbit IgG (H + L) Cross-Adsorbed Secondary Antibody, Alexa Fluor 488 (Thermo Fisher Scientific) was used. After the reaction, sections were incubated with BBT and washed with TBS-T. The negative control was labeled with only the secondary antibody, and all other procedures were performed as described above. As a contrasting stain, a 4′,6-diamidino-2-phenylindole (DAPI) solution (1 mg/mL) (Nacalai tesque, Kyoto, Japan) was prepared at a concentration of 0.1 mg/ml and reacted with the sections for 30 min in the dark at room temperature. Sections were then washed with TBS-T and mounted using VECTASHIELD Mounting Medium (Vector Laboratories, CA, USA). Sections were observed under a Biozero microscope (Keyence, Osaka, Japan).

## Results

### RT-qPCR analysis of RS008881

**Fig 1:**
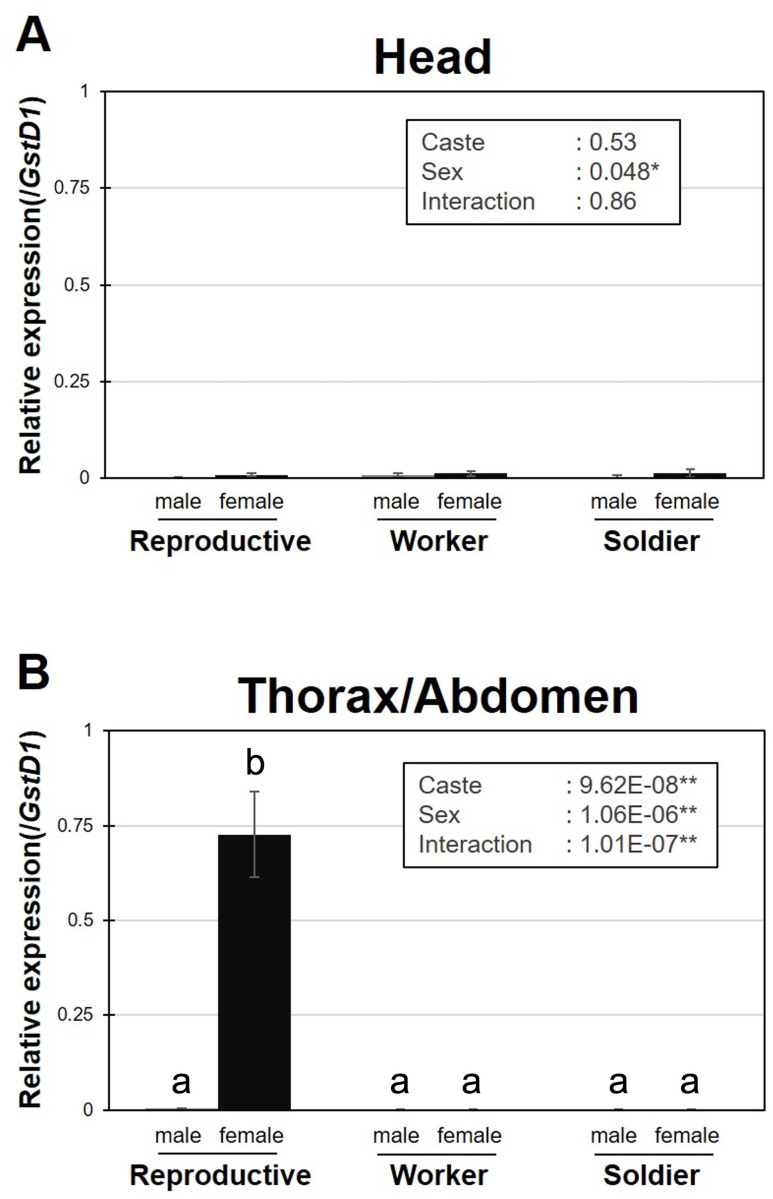
Real-time qPCR expression levels (mean ± S.D., biological triplicates) of *RS008881* in the head (A) and thorax/abdomen (B) among primary reproductives, workers, and soldiers. Total RNA was extracted from three individuals per sample from each colony and three biological samples (derived from different colonies) were prepared. Each value was normalized to the expression levels of *GstD1* (**Table 2**). Statistical results of two-way ANOVA are described in each box (*P < 0.05, **P < 0.01). Different letters above the bars indicate significant differences (two-way ANOVA followed by Tukey’s test; *P* < 0.05). qPCR, quantitative polymerase chain reaction; ANOVA, analysis of variance.

We investigated the expression patterns of *RS008881* among different castes (female/male primary reproductives, soldiers, and workers; heads and thorax/abdomen parts) using RT-qPCR. *GstD1* was selected as an internal reference gene using GeNorm and NormFinder (**Table 2**). High expression levels of *RS008881* were observed in the thorax/abdomen of female primary queens, consistent with the results of a previous study using RNA-seq analysis [2] (**Fig 1 and S1 Table**).

**Table 2:** Stability values of reference genes in real-time qPCR analysis among castes.

Gene symbol	Accession no/Gene ID	Stability value	
		GeNorm	NormFinder
*EF-1*	AB602838	0.708	0.392
*NADH-dh*	AB602837	0.574	0.101
*β-Actin*	AB520714	1.099	0.741
GstD1*	RS001168	0.572	0.075
*EIF-1*	RS005199	0.556	0.132
*RPS18*	RS015150	0.725	0.424

*GstD1 was selected by GeNorm and NormFinder due to the low stability values among six genes analyzed.

**Fig 2:**
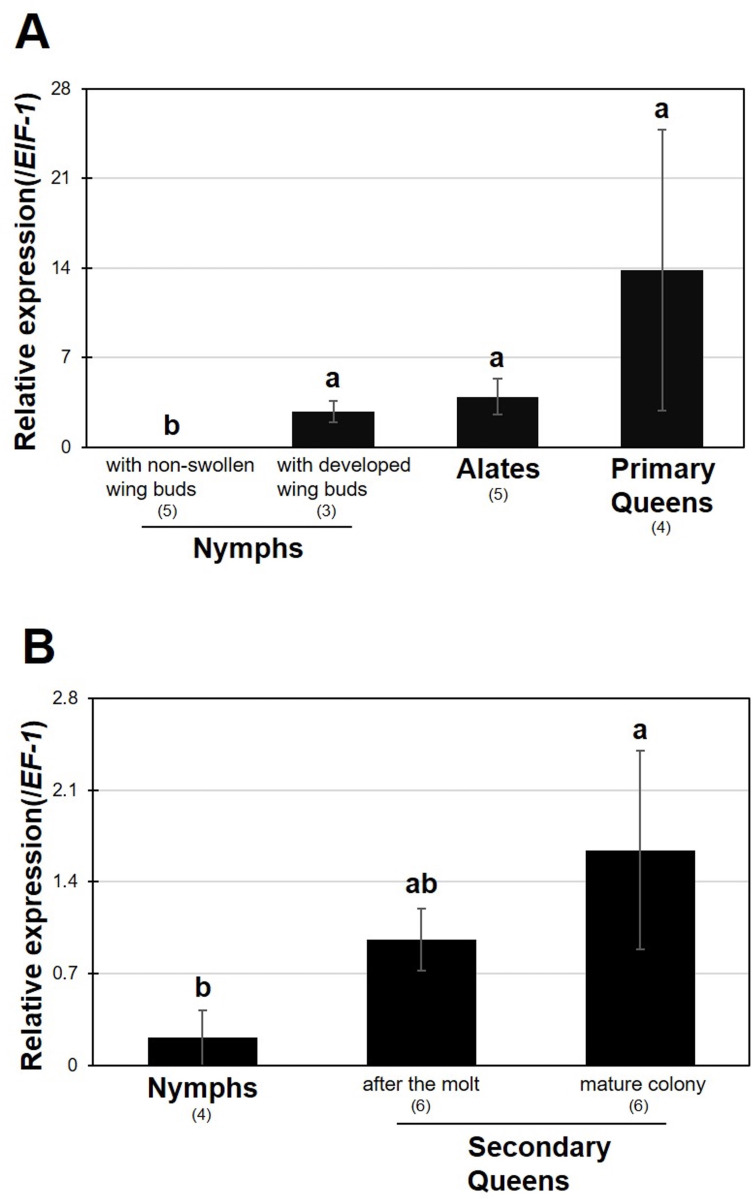
Real-time qPCR expression levels (mean ± S.D., biological replications = 3–6) of *RS008881* in various developmental stages during the formation of primary queens (A) and secondary queens (B). Total RNA was extracted from abdomens of two individuals per each sample. Each value is normalized to the expression levels of *EIF-1* (A) or *EF-1* (B) (**Tables 3** and **4**). Different letters above the bars indicate significant differences (one-way ANOVA followed by Tukey-Kramer test; *P* < 0.05). qPCR, quantitative polymerase chain reaction; ANOVA, analysis of variance.

Using female individuals, we compared the *RS008881* expression levels at various developmental stages. To compare the various developmental stages during the formation of primary female reproductives (nymphs, alates, and primary queens), *ElF-1* was selected as the internal reference gene (**Table 3**). Significantly higher expression levels were observed in primary queens, alates, and last-instar nymphs with fully developed wing buds than in the earlier stages (last-instar nymphs with non-swollen developed wing buds) (**Fig 2A and S1 Table**). For comparing developmental stages of secondary female reproductives (sixth-instar nymphs, secondary queens within 24 h after molting, and physogastric secondary queens collected from a mature colony), *EF-1* was selected as the internal reference gene (**Table 4**). Gene expression levels of the physogastric queens were significantly higher than those of the sixth-instar nymphs (**Fig 2B and S1 Table**).

**Table 3:** Stability values of reference genes in real-time qPCR analysis among various developmental stages during the formation of primary queens.

Gene symbol	Accession no/Gene ID	Stability value	
		GeNorm	NormFinder
*EF-1*	AB602838	0.595	0.349
*NADH-dh*	AB602837	0.501	0.162
*β-Actin*	AB520714	0.832	0.546
*GstD1*	RS001168	0.542	0.215
EIF-1*	RS005199	0.429	0.106
*RPS18*	RS015150	0.516	0.255

*EIF-1 was selected by GeNorm and NormFinder due to the lowest stability values among six genes analyzed.

**Table 4:** Stability values of reference genes in real-time qPCR analysis among various developmental stages during the formation of secondary queens.

Gene symbol	Accession no/Gene ID	Stability value	
		GeNorm	NormFinder
EF-1*	AB602838	0.359	0.071
*NADH-dh*	AB602837	0.408	0.103
*β-Actin*	AB520714	0.869	0.589
*GstD1*	RS001168	0.394	0.202
*EIF-1*	RS005199	0.387	0.188
*RPS18*	RS015150	0.480	0.221

*EF-1 was selected by GeNorm and NormFinder due to the lowest stability values among six genes analyzed.

### Western blotting of RS008881 products

**Fig 3:**
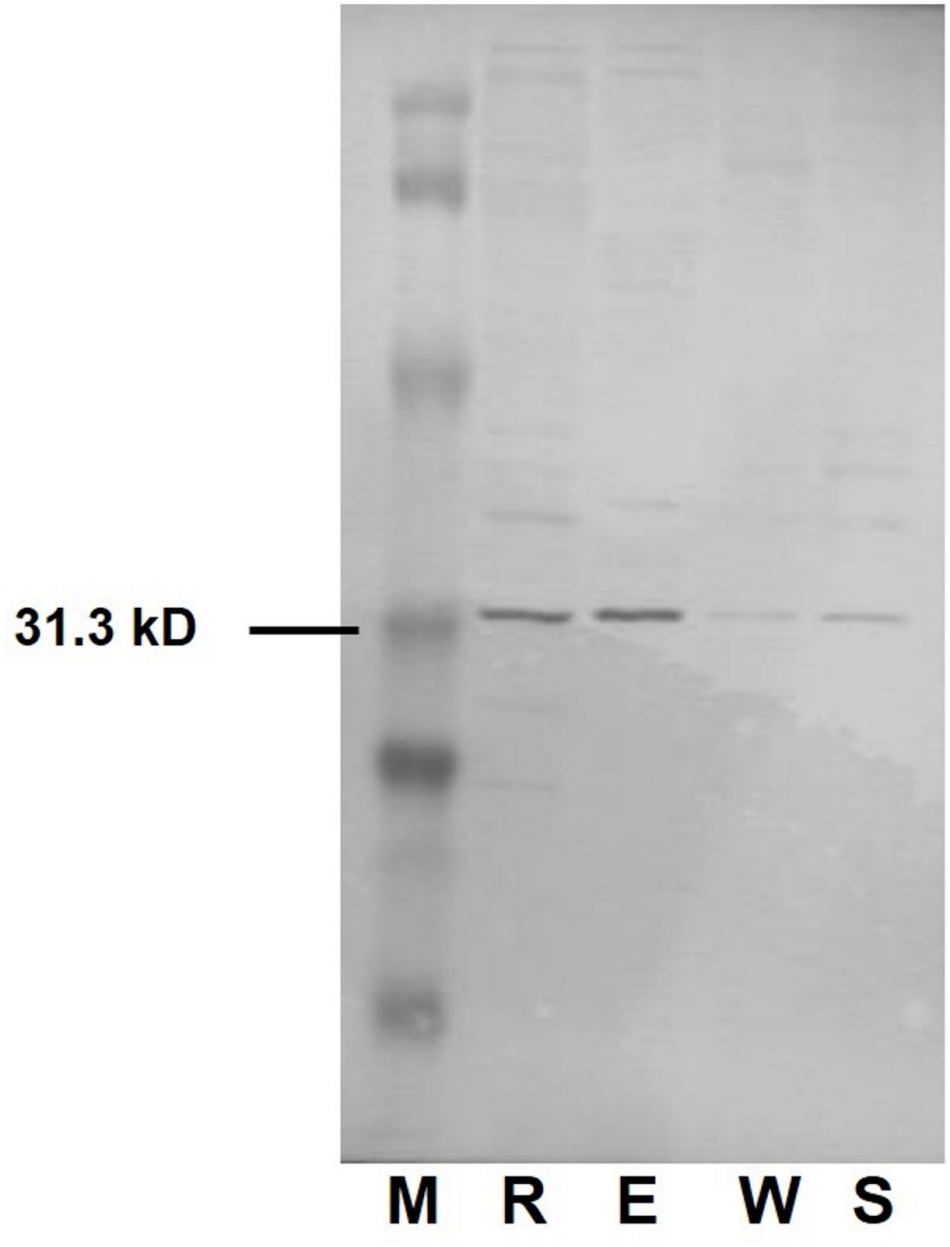
Western blotting analysis of RS008881 protein. The proteins extracted from whole bodies (five individuals) of female secondary reproductives (R), workers (W), soldiers (S), and egg masses (approximately 25 mg) obtained from a mature colony (E). A clear band of approximately 31.3 kD was observed in the R and E lanes.

**Fig 4:**
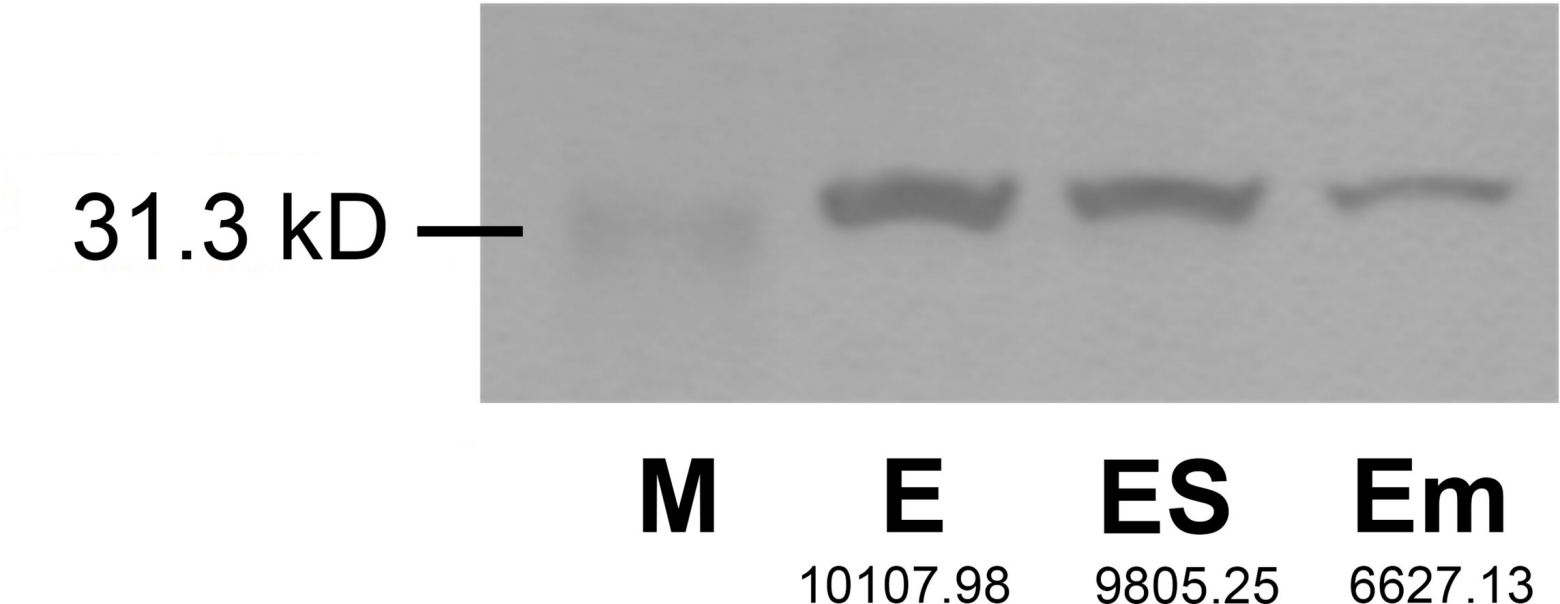
Western blotting analysis of RS008881 protein in eggs. Proteins extracted from egg masses (approximately 25 mg) (E), eggshells (ES), and the liquid fraction containing embryos (Em). A clear band of approximately 31.3 kD was observed in all lanes. Calculated signal intensity (luminance value) is indicated in each lane.

Western blotting was performed using affinity-purified antibodies against protein extracts obtained from secondary queens, workers, soldiers, and egg masses. In particular, strong signals were observed in the protein extracts from secondary queens and egg masses (**Fig 3 and S1 Raw images**). Next, egg masses collected from mature colonies were separated into eggshells and the liquid fraction containing embryos. Protein extracts from each fraction were subjected to western blotting. The results showed a strong signal in the eggshell protein extracts (**Fig 4 and S1 Raw images**). After normalization to total protein, we observed that the signal intensity of the protein extracted from the eggshell (luminance value: 9805.25) was approximately 1.5 times stronger than that of the liquid fraction containing the embryos (luminance value: 6627.13).

### Immunohistochemistry of RS008881 products

**Fig 5:**
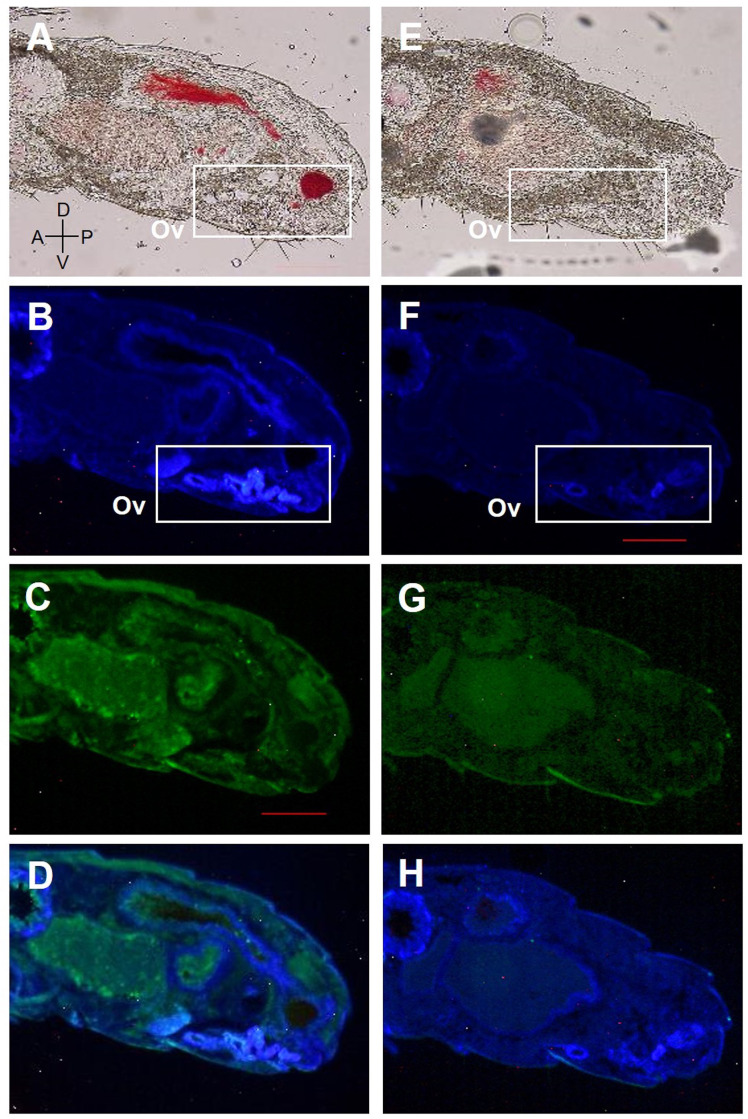
**RS008881 protein localization in a sagittal section (10 μm) of the abdomen of female 8th instar nymphs.** The panels (A)–(D) and (E)–(H) indicate the same section. The sagittal section using bright field microscopy (A, E). DAPI staining without a RS008881 primary antibody (B, F). RS008881 immunostaining with RS008881 primary and secondary antibodies (C). RS008881 immunostaining without a RS008881 primary antibody (only with secondary antibody) (G). DAPI staining and RS008881 immunostaining with RS008881 primary and secondary antibodies (D). DAPI staining and RS008881 immunostaining without a RS008881 primary antibody (only with secondary antibody) (H). Ovary (Ov). Scale bars indicate 300 μm. DAPI, 4′,6-diamidino-2-phenylindole.

**Fig 6:**
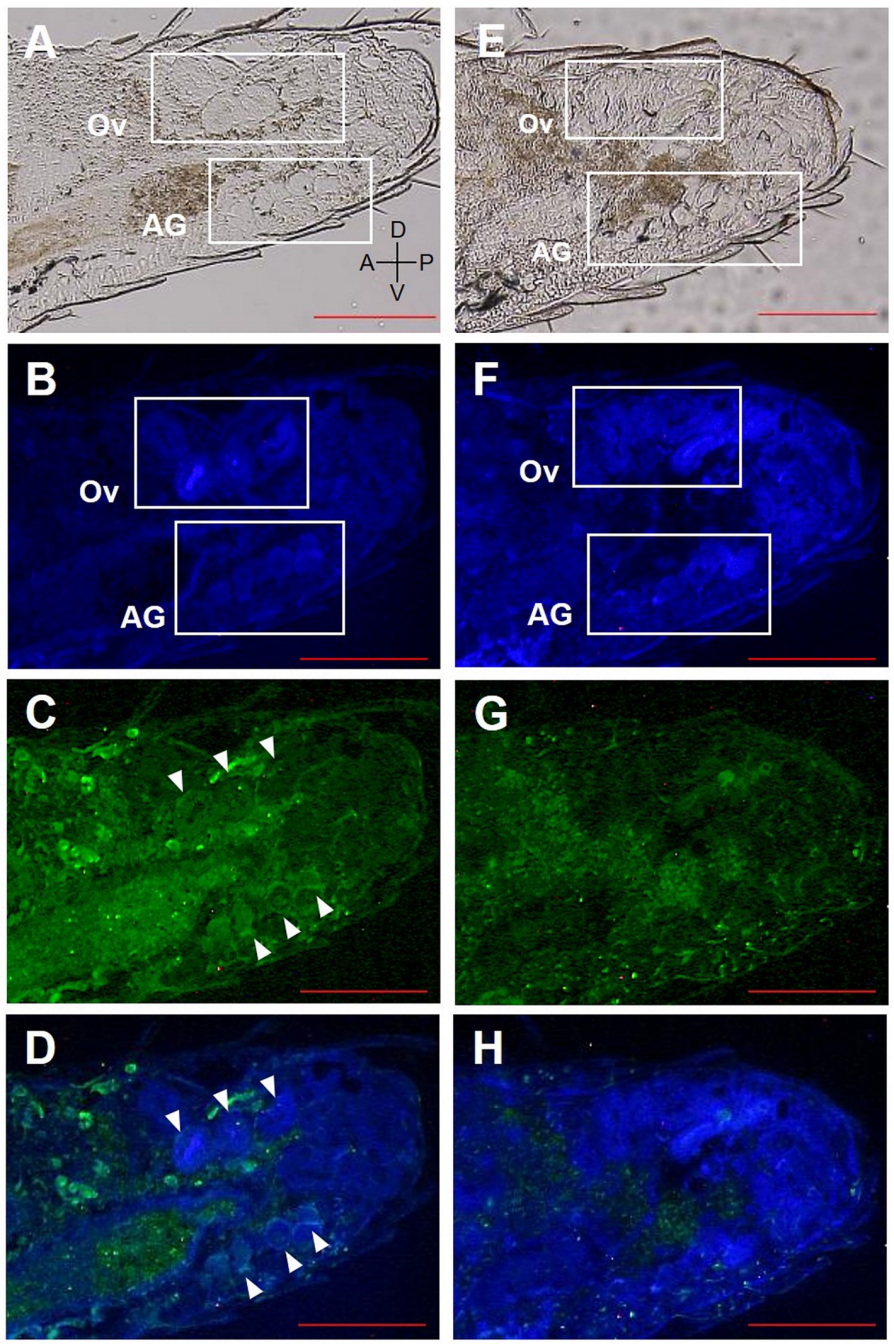
RS008881 protein localization in a sagittal section (10 μm) of the abdomen of female primary reproductives. The panels (A)–(D) and (E)–(H) indicate the same section. The sagittal section using bright field microscopy (A, E). DAPI staining without a RS008881 primary antibody (B, F). RS008881 immunostaining with RS008881 primary and secondary antibodies (C). RS008881 immunostaining without a RS008881 primary antibody (only with secondary antibody) (G). DAPI staining and RS008881 immunostaining with RS008881 primary and secondary antibodies (D). DAPI staining and RS008881 immunostaining without a RS008881 primary antibody (only with secondary antibody) (H). Ovary (Ov). Scale bars indicate 300 μm. DAPI, 4′,6-diamidino-2-phenylindole.

**Fig 7:**
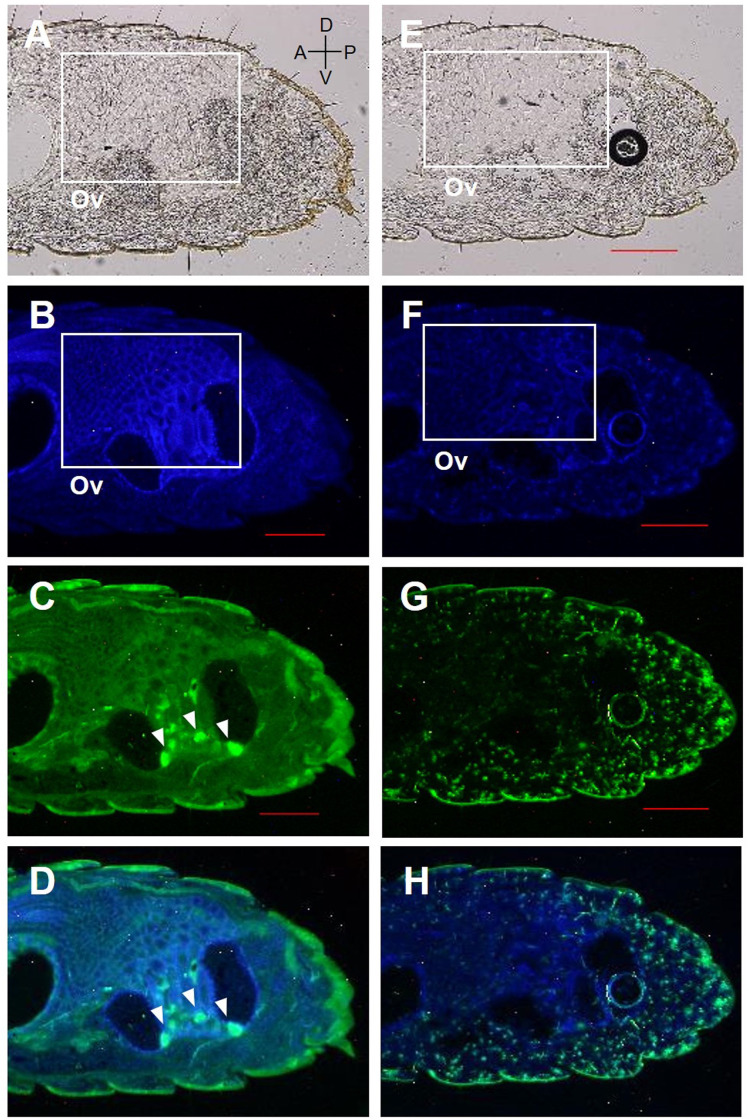
RS008881 protein localization in a sagittal section (10 μm) of the abdomen of female secondary reproductives. The panels (A)–(D) and (E)–(H) indicate the same section. The sagittal section using bright field microscopy (A, E). DAPI staining without a RS008881 primary antibody (B, F). RS008881 immunostaining with RS008881 primary and secondary antibodies (C). RS008881 immunostaining without a RS008881 primary antibody (only with secondary antibody) (G). DAPI staining and RS008881 immunostaining with RS008881 primary and secondary antibodies (D). DAPI staining and RS008881 immunostaining without a RS008881 primary antibody (only with secondary antibody) (H). Ovary (Ov). Scale bars indicate 300 μm. DAPI, 4′,6-diamidino-2-phenylindole.

**Fig 8:**
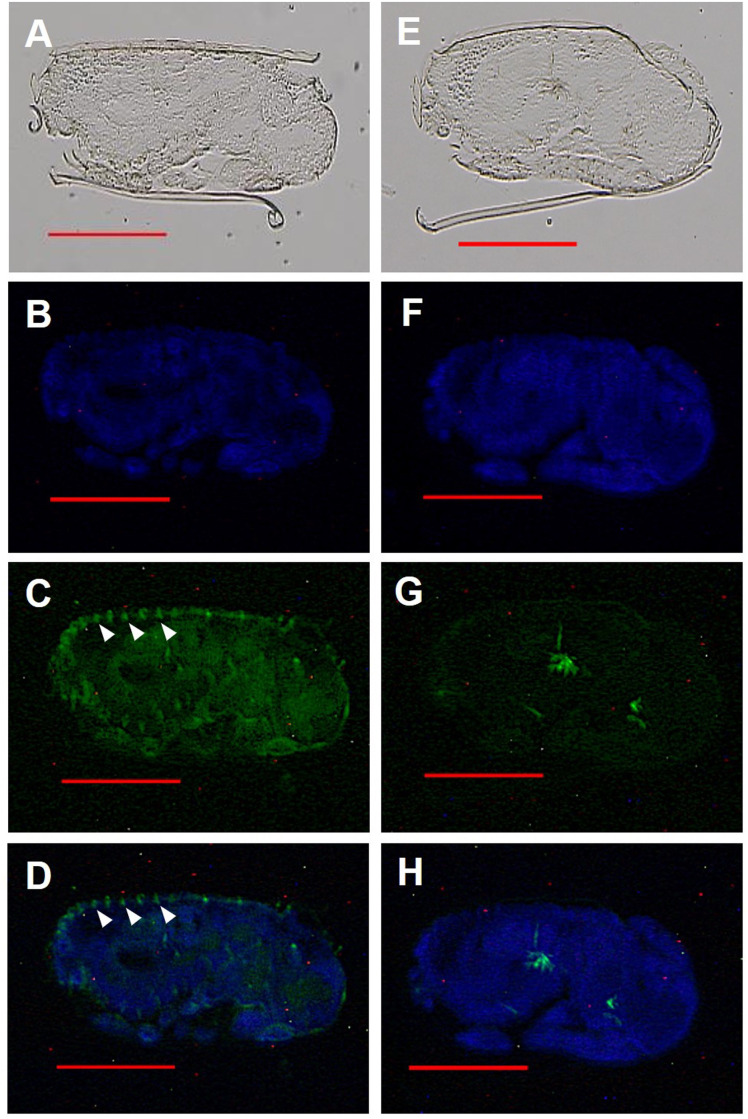
RS008881 protein localization in a longitudinal section (10 μm) of eggs obtained from a mature colony. The panels (A)–(D) and (E)–(H) indicate the same section. The sagittal section using bright field microscopy (A, E). DAPI staining without a RS008881 primary antibody (B, F). RS008881 immunostaining with RS008881 primary and secondary antibodies (C). RS008881 immunostaining without a RS008881 primary antibody (only with secondary antibody) (G). DAPI staining and RS008881 immunostaining with RS008881 primary and secondary antibodies (D). DAPI staining and RS008881 immunostaining without a RS008881 primary antibody (only with secondary antibody) (H). Scale bars indicate 300 μm. DAPI, 4′,6-diamidino-2-phenylindole.

Fluorescent immunohistochemistry was performed using affinity-purified antibodies on the abdomen of female reproductives, last-instar nymphs, and eggs (**Figs 5–8**). In the analysis of last-instar nymphs, no specific signals were observed in the cells stained with DAPI (**Fig 5D**). In contrast, in the analysis using primary queens, specific signals were detected in the accessory glands of the ovaries and ovarian tubules (**Fig 6C and 6D**). In secondary queens, particularly strong signals were observed near the ends of the ovarian tubules containing developed oocytes (**Fig 7C and 7D**). Furthermore, when eggs collected from mature colonies were analyzed, signals were observed on the eggshells (**Fig 8C and 8D**).

## Discussion

### Lipocalin gene expression patterns

The expression levels of the lipocalin gene *RS008881* were compared among the castes (primary reproductives, workers, and soldiers) in their heads and bodies (thorax and abdomen) using RT-qPCR. The results revealed high expression levels in the bodies of primary female reproductives (queens). Furthermore, expression analysis during the female reproductive caste differentiation showed that *RS008881* was expressed not only in primary and physogastric secondary queens, but also in winged imagoes (alates) and secondary queens just after molting. Additionally, the expression was confirmed in last-instar nymphs immediately before molting into alates. These results indicate that *RS008881* is not specifically expressed in female primary queens but is highly expressed in female reproductives with mature eggs. To investigate its relationship with the reproductive cycle of primary queens [13], expression analyses in primary queens at different colony developmental times is required.

### Potential roles of lipocalin RS008881

The lipocalin gene *RS008881* is highly expressed in primary queens and its mRNA is localized in the ovarian accessory gland cells [2]. Immunohistochemical staining revealed that RS008881 products were localized in the ovarian tubules and accessory glands of both primary and secondary queens, consistent with the results of gene expression analysis. Especially, strong signals were observed at the distal ends of ovarian tubules in secondary queens. However, in the last-instar nymph stage, while immature eggs were observed within the ovarian tubules, specific signals were not detected. Furthermore, the results of immunohistochemical staining of eggs and western blotting using eggshell extracts showed strong signals in the eggshells. These results suggest that *RS008881* products are associated with mature eggs and localized in eggshells following oviposition. In several cockroach species, including the sister group of termites (genus *Cryptocercus*) and primitive termite lineages, such as *Mastotermes darwiniensis* and *Zootermopsis nevadensis*, substances secreted from the ovarian glands have been suggested to contribute to egg case (or similar structures) formation and the adhesion of eggs to one another [4, 21]. Therefore, *RS008881* products secreted from the ovarian accessory glands may play a role in eggshell structure formation and physical protection of embryos by the eggshell.

Furthermore, a previous study showed that odorant-binding proteins (OBPs) are structural components of eggshells in the yellow fever mosquito *Aedes aegypti* [22]. Insect OBPs are well-known for their general role in chemical substance transport [23, 24]. Although lipocalins and OBPs have different three-dimensional structures, they share high stability and versatility and possess common functions, such as pheromone transport [25]. In *R*. *speratus*, volatile compounds (n-butyl-n-butyrate and 2-methyl-1-butanol), probably released from female reproductives and eggs, reportedly act as pheromones regulating the caste differentiation of new reproductives [12]. Additionally, β-glucosidase presented on the surface of eggs has been shown to function as an egg recognition pheromone for workers [11]. A paralog of β-glucosidases was reported to be strongly expressed in the ovarian accessory glands of primary queens in *R*. *speratus* [2]. Consequently, we suggest that the *RS008881* products serve as carriers of these pheromonal substances. Further functional analyses are necessary to investigate the effect of RS008881 on the external structure of eggs, hatching rate, egg recognition by workers, and caste differentiation.

Lipocalins are known to have diverse roles in animals [6]. Based on the expression analysis of RNA-seq data [2], out of the 18 known lipocalin genes in *R*. *speratus*, *RS008881* and *RS008884* are specifically highly expressed in queens. In this study, a single PCR product was confirmed by the dissociation curve using the specific primers and protein localization was verified using an RS008881-specific antibody, allowing us to infer that the predicted functions are attributed to RS008881. However, if the three-dimensional structure of RS008881 changes (e.g., due to ligand binding), the signal strength may vary. Further detailed analysis is needed to determine the precise protein localization and movement of RS008881.

## Conclusions

We investigated the lipocalin gene *RS008881*, which is highly expressed in the ovarian accessory glands of the primary reproductives in *R*. *speratus* [2]. RT-qPCR analysis revealed the elevated expression of *RS008881* during the formation of both primary and secondary queens. Moreover, protein analysis utilizing specific peptide antibodies demonstrated the localization of *RS008881* products in the eggshells of produced eggs and ovaries of female reproductives. *RS008881* may play a dual role, not only in physically protecting eggs but also in serving as a carrier for pheromonal substances derived from female reproductives. To clarify this possibility, RNA interference (RNAi) should be performed on pre-oviposition queens. The survival rate and hatchability of the resulting eggs, as well as the pheromonal effects on individuals, should then be investigated.

## Supporting information

S1 Table RT-qPCR data for Figs 1 and 2. (XLSX)

S1 Raw images Representative original blots for Figs 3 and 4. (PDF)
