# Supplementary material for: Protein localization and potential function of lipocalin in Reticulitermes speratus queens
Source: PLoS One. 2024 Oct 7;19(10):e0311836. doi: 10.1371/journal.pone.0311836 (PMC11458055; doi:10.1371/journal.pone.0311836)
Supplement: S1 Raw images — (PDF) [file pone.0311836.s002.pdf]

Representative original blot to protein expression of RS008881 (Fig. 3)

Protein ladder

114 kD
94.7 kD
47.3 kD
31.3 kD
25.7 kD
17.4 kD

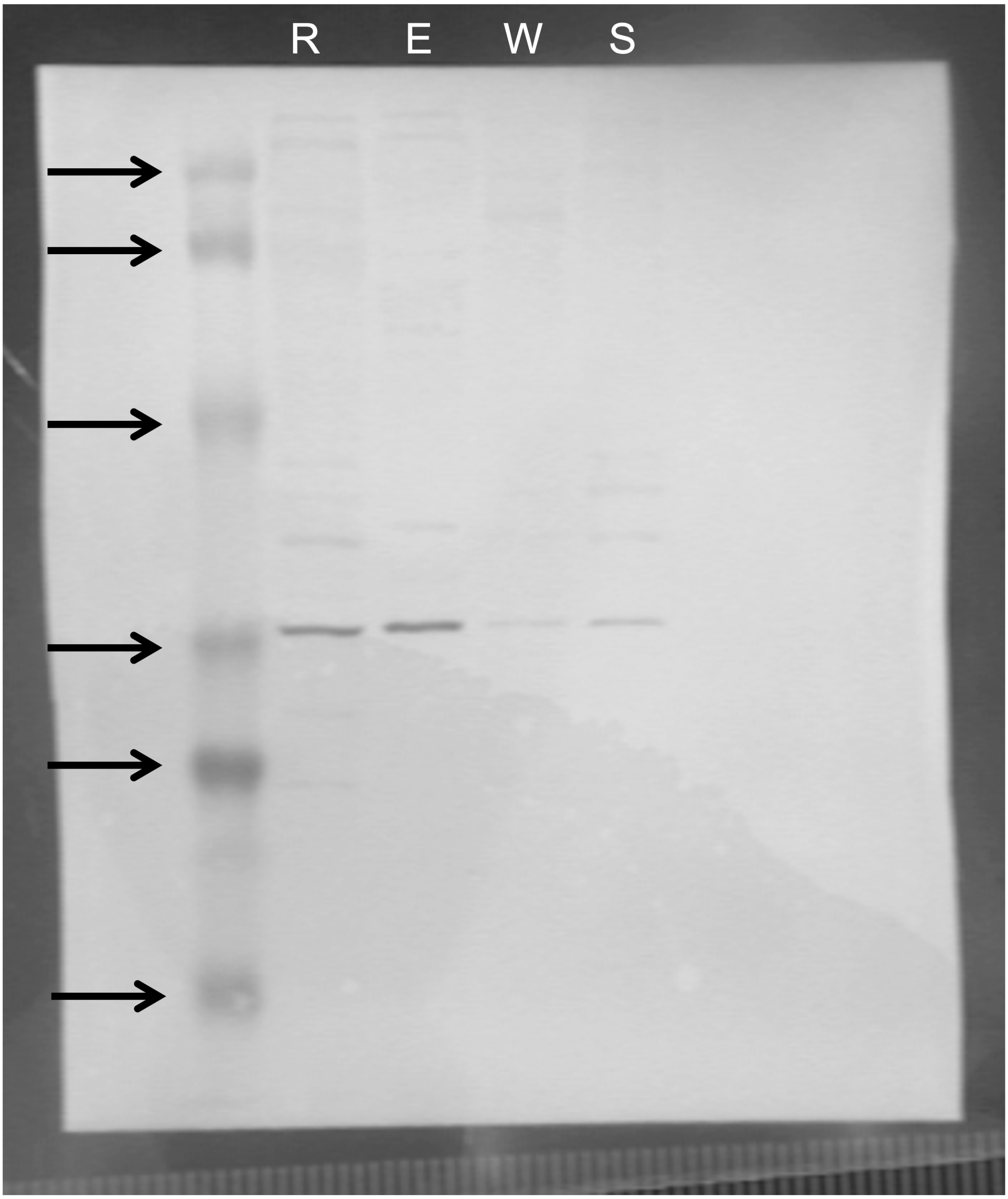

R: female secondary reproductives
E: egg masses, W: workers, S: soldiers

Representative original blot to protein expression of RS008881 (Fig. 4)

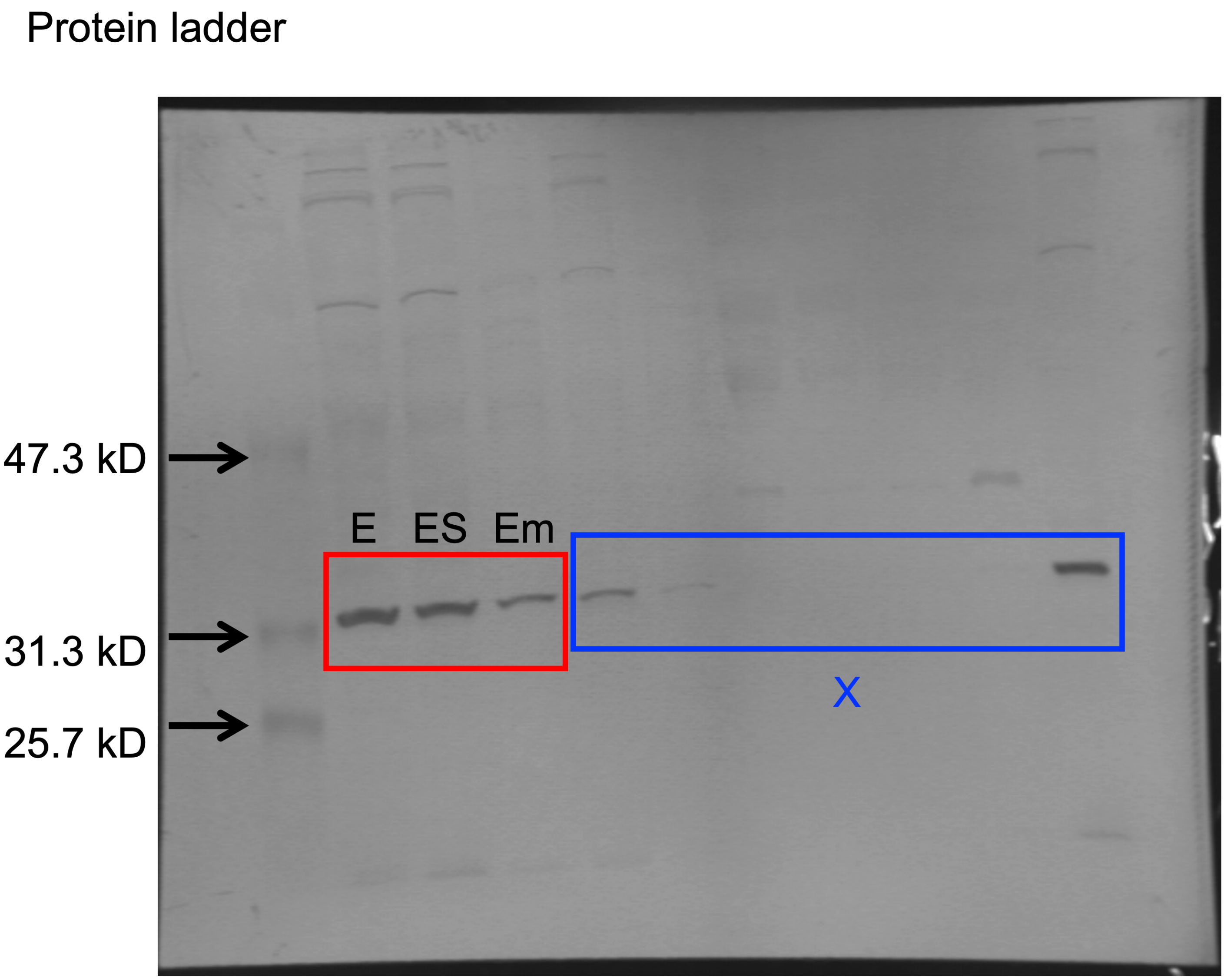

E: egg masses
ES: eggshells
Em: liquid fraction containing embryos

[red box] lanes included in the final figure

[blue box] lanes not included in the final figure
X
